# Performance Optimization of an Air-Standard Irreversible Dual-Atkinson Cycle Engine Based on the Ecological Coefficient of Performance Criterion

**DOI:** 10.1155/2014/815787

**Published:** 2014-07-06

**Authors:** Guven Gonca, Bahri Sahin

**Affiliations:** Department of Naval Architecture and Marine Engineering, Yildiz Technical University, Besiktas, 34349 Istanbul, Turkey

## Abstract

This paper presents an ecological performance analysis and optimization for an air-standard irreversible Dual-Atkinson cycle (DAC) based on the ecological coefficient of performance (ECOP) criterion which includes internal irreversibilities, heat leak, and finite-rate of heat transfer. A comprehensive numerical analysis has been realized so as to investigate the global and optimal performances of the cycle. The results obtained based on the ECOP criterion are compared with a different ecological function which is named as the ecologic objective-function and with the maximum power output conditions. The results have been attained introducing the compression ratio, cut-off ratio, pressure ratio, Atkinson cycle ratio, source temperature ratio, and internal irreversibility parameter. The change of cycle performance with respect to these parameters is investigated and graphically presented.

## 1. Introduction

In the recent years, the studies related to engine research focused on reducing pollutant emissions, particularly NO_*x*_, released from internal combustion engines owing to environmental regulations and restrictions. The application of the Atkinson and Miller cycles to the internal combustion engines (ICE) may reduce NO_*x*_ emissions with little cost of power [1–11]. In the literature, various optimization studies on the Miller and Atkinson cycles have been carried out. However, it should be noted that, at the same peak combustion temperatures and pressures, Atkinson cycle could become more efficient than the Miller cycle due to higher expansion ratio, as the amount of heat wasted with the exhaust gases may be reduced and transformed to power output by increasing expansion ratio. There are various studies on the Miller and Atkinson cycles. Gonca et al. [1–5] showed that the Miller cycle diesel engine is more advantageous than conventional diesel engine in terms of NO emissions and effective efficiency depending on numerical studies. Wang et al. experimentally [6] and analytically [7] implemented the Miller cycle into a petrol engine in order to decrease the NO_*x*_ emissions. Mikalsen et al. [8] applied the Miller cycle into an Otto cycle natural gas engine and the SFC and power output of the engine decreased. Kesgin [9] experimentally and theoretically applied the Miller cycle into a natural gas engine and the efficiency increased and NO_*x*_ emissions could be abated. Wang et al. [10] experimentally applied the Miller cycle into a diesel engine and NO_*x*_ emissions decreased. Lin and Hou [12] expressed that the performance of Miller cycle is higher than that of Otto cycle at same peak temperature conditions. The influences of temperature-dependent specific heats of the working fluid on the performance characteristics were investigated for an air-standard reversible Miller cycle [13] and irreversible Miller cycle with different specific heat models [14]; the total cycle volumes and pressure ratios of the Miller cycle were depicted with graphics at maximum power density conditions [15] by Al-Sarkhi et al., and Zhao and Chen [16] analyzed the performance of an air-standard irreversible Miller cycle by introducing the pressure ratios and considering the irreversibilities during the cycle processes. Wang et al. [17] experimentally applied the Miller cycle into a diesel engine. Wu et al. [18] theoretically applied the Miller cycle into a supercharged Otto engine. Ebrahimi [19, 20] conducted thermodynamical analyses for reversible Miller cycle with considerations of engine speed and variable specific heat ratio of working fluid [19] and for irreversible Miller cycle with respect to the variation of relative air-fuel ratio and stroke length [20]. Wang and Hou [21] conducted a performance analysis for an Atkinson cycle coupled with variable temperature heat reservoirs under maximum power (MP) and maximum power density (MPD) conditions. Chen et al. [22] optimized the air-standard Atkinson cycle based on the MPD criterion. Al-Sarkhi et al. [23] expanded the study in [22] using temperature-dependent specific heat model and it was emphasized that this model has substantial influence on the performance of the Atkinson cycle. Ust [24] conducted a performance analysis and optimization for the irreversible Atkinson cycle by considering the internal irreversibilities originating from the adiabatic compression and expansion processes in order to define the optimum performance and design parameters of the cycle. Zhao and Chen [25] analyzed an irreversible Atkinson cycle by taking account of irreversibilities originating from the adiabatic processes, finite-time processes, and heat transfer through the cylinder wall. Gahruei et al. [26] compared the performances of the classical Dual and Dual-Atkinson cycles based on finite-time thermodynamics by considering variable specific heats of the working fluid, heat transfer, and friction losses. Ge et al. [27] carried out a performance optimization for an endoreversible Atkinson heat engine. Ge et al. [28] investigated the influences of variable specific heats of the working fluid on the performance of Atkinson cycle. Zhao et al. [29] performed an experimental and numerical study to design and optimize an Atkinson cycle engine by using Artificial Neural Network Method. Zhao and Xu [30] improved the fuel economy of an Atkinson cycle engine up to 7.67% by using the Genetic Algorithm. Ebrahimi [31] carried out a performance optimization of an Atkinson cycle heat engine by taking into account the impacts of the cylinder wall temperature, mean piston velocity, and equivalence ratio. Lin and Hou [32] examined the impacts of variable specific heats of the working fluid, friction, and losses, as a percentage of fuel's energy, on the performance of an air-standard Atkinson cycle. Hou [33] compared the performances of the air-standard Otto and Atkinson cycles by taking into consideration the heat transfer impacts.

The studies related to performance optimization and thermodynamical analyses of engine cycles were realized with various methods and objective functions. One of the objective functions commonly used is the ecological objective-function proposed by Angulo-Brown [34]. This function is determined as the power output minus the loss rate of availability. In recent years, a new thermoecological objective-function has been developed by Ust et al. [35, 36]. This objective-function is called the ecological coefficient of performance (ECOP), which is stated as the proportion of the power output to the loss rate of availability. It was asserted that the ECOP criterion is more understandable compared to ecological objective-function [36], as the minimum entropy is formed at the maximum ECOP conditions.

Various studies have been performed by applying the ECOP criterion to the heat engines [37–43]. Ust et al. [36] carried out an ecological performance analysis for an irreversible dual cycle based on the ECOP criterion considering finite-rate of heat transfer, heat leak, and internal irreversibilities. Ust et al. [37–40] carried out performance analyses and optimizations for irreversible Carnot heat engine [37] and Brayton heat engine [38–40] considering losses owing to heat leak, heat transfer, and internal irreversibilities, based on ecological coefficient of performance (ECOP) function. Sogut et al. [41] investigated the influences of intercooling and regeneration on the thermoecological performance analysis of an irreversible-closed Brayton heat engine with variable temperature thermal reservoirs. Ust and Sahin [42] carried out a performance optimization for irreversible refrigerators based on the ECOP criterion. Ust [43] carried out a performance analysis based on ECOP criterion for irreversible air refrigeration cycles considering irreversibilities because of finite-rate heat transfer, heat leakage, and internal dissipations. This study presents a thermoecological performance analysis based on the ECOP, the ecological objective-function (*$\dot{E}$*), and the maximum power output conditions for an irreversible DAC engine. The influences of the engine design parameters on the engine performance were examined. The general and optimal design parameters which give the maximum ECOP, the maximum *$\dot{E}$*, and the maximum power output have been computationally determined. In the literature, there is no such study which applies the *$\dot{E}$* and ECOP function on an irreversible DAC engine. Therefore, this study could be used as a guideline by real engine designers to obtain maximum ecological performance for DAC engines.

## 2. Theoretical Analysis of DAC

**Figure 1:**
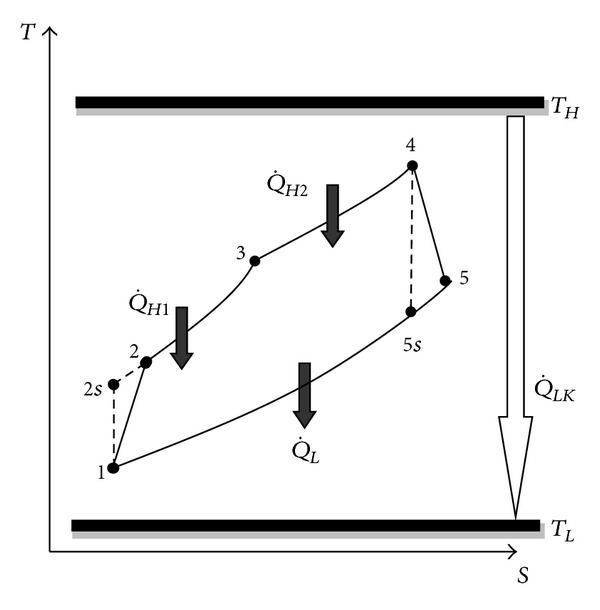
*T*-*S* schematic diagram for DAC.

*P*-*V* and *T*-*S* diagrams of the irreversible air-standard DAC (1-2-3-4-5-1) coupled with constant hot and cold temperature heat-reservoirs are depicted in Figure 1. It is clear that the process 1-2 is an irreversible compression and internal irreversibilities were taken into account, whilst the process 1-2 s is an isentropic compression. The heat input is provided in the processes 2-3 (at constant volume) and 3-4 (at constant pressure). The process 4-5 is an irreversible expansion and internal irreversibilities were considered, while the process 4-5 s is an isentropic expansion. The heat rejection occurs in the process 5–1 (at constant pressure) and the cycle is completed.

In DAC cycle, ${\dot{Q}}_{H1}$ and ${\dot{Q}}_{H2}$ are the heat transfer rates (time-dependent) from the hot resource at temperature *T*_*H*_ to the working fluid (ideal air) in the processes 2-3 and 3-4; ${\dot{Q}}_{L}$ is the heat transfer rate from the working fluid to the cold-reservoir at temperature *T*_*L*_ in the process 5–1. ${\dot{Q}}_{H1}$, ${\dot{Q}}_{H2}$, and ${\dot{Q}}_{L}$ are expressed by expanding Ust et al.'s study [36] as follows:

(1) Q˙H1= UH1AH1(TH−T2)−(TH−T3)ln⁡⁡((TH−T2)/(TH−T3))= C˙WεH1(TH−T2)=C˙W(T3−T2),

(2) Q˙H2=UH2AH2(TH−T3)−(TH−T4)ln⁡⁡((TH−T3)/(TH−T4))=kC˙WεH2(1−εH1)(TH−T2)=kC˙W(T4−T3),

(3) Q˙L=ULAL(T5−TL)−(T1−TL)ln⁡⁡((T5−TL)/(T1−TL))=kC˙WεL(1−εL)(T5−TL)=kC˙W(T5−T1),

(4) C˙W=m˙CV,

where *U*_*H*1_ *A*_*H*1_, *U*_*H*2_ *A*_*H*2_, and *U*_*L*_ *A*_*L*_ are the conductance of the hot-reservoir at constant volume and at constant pressure and the conductance of cold-reservoir heat exchanger, respectively. ${\dot{C}}_{W}$ is the capacity of the working fluid and *k* is the isentropic exponent that is stated as the ratio of the specific heat at constant pressure to the specific heat at constant volume (*C*_*P*_/*C*_*V*_) of working fluid. *ε*_*H*1_, *ε*_*H*2_, and *ε*_*L*_ are the effectiveness of the hot- and cold-reservoirs of the heat exchanger which are expressed as below:

(5) εH1=1−exp⁡(−NH1);εH2=1−exp⁡(−NH2);εL=1−exp⁡(−NL),

where *N*_*H*1_, *N*_*H*2_, and *N*_*L*_ are the number of heat transfer units for hot-side and cold-side based on the minimum thermal capacity rates and they could be given as below:

(6) $$\begin{array}{c} N_{H1}=\frac{U_{H1}A_{H1}}{{\dot{C}}_{W}};\;\;N_{H2}=\frac{U_{H2}A_{H2}}{k{\dot{C}}_{W}};\\ N_{L}=\frac{U_{L}A_{L}}{{\dot{C}}_{W}}. \end{array}$$

The heat leakage rate, ${\dot{Q}}_{LK}$, from the hot source at temperature *T*_*H*_ to the cold source at temperature *T*_*L*_ could be written as follows:

(7) $$\begin{array}{c} {\dot{Q}}_{LK}={\dot{C}}_{I}\left(T_{H}-T_{L}\right)=\xi {\dot{C}}_{W}\left(T_{H}-T_{L}\right), \end{array}$$

where ${\dot{C}}_{I}$ is the internal conductance of the DAC and *ξ* is the percentage of the internal conductance with respect to the thermal capacity rate of the working fluid. The total heat rate, ${\dot{Q}}_{HT}$, transferred from hot-side is written as

(8) Q˙HT=C˙W{[εH1+kεH2(1−εH1)](TH−T2)+ξ(TH−TL)},

and the total heat rate, ${\dot{Q}}_{LT}$, transferred to the cold-side is expressed as

(9) $$\begin{array}{c} {\dot{Q}}_{LT}={\dot{C}}_{W}\left\{\left[\varepsilon_{L1}+k\varepsilon_{L2}\left(1-\varepsilon_{L1}\right)\right]\left(T_{5}-T_{L}\right)+\xi \left(T_{H}-T_{L}\right)\right\}. \end{array}$$

We can obtain below equations using (1)–(3):

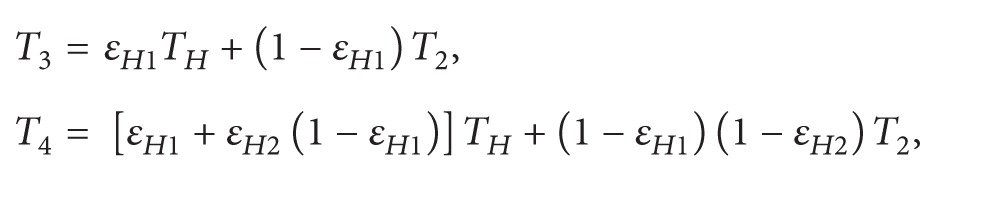
(10)

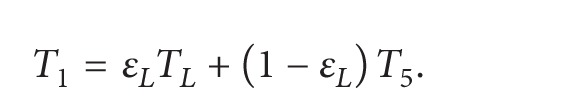
(11)
The net power output may be stated by using the first law of thermodynamics:

(12) W˙=(Q˙H1+Q˙H2)−Q˙L,W˙=C˙W{[εH1+kεH2(1−εH1)](TH−T2)−kεL(1−εL)(T5−TL)}.

From (13),

(13) T5=TL+[εH1+kεH2(1−εH1)](TH−T2)kεL(1−εL) −W˙C˙WkεL(1−εL).

Substituting (13) into (11) we obtain the equation as below:

(14) T1=εLTL+(1−εL) ×[TL+[εH1+kεH2(1−εH1)](TH−T2)kεL(1−εL)−W˙C˙WkεL(1−εL)].

The isentropic efficiencies of the compression and expansion processes are given as below [5]:

(15) $$\begin{array}{c} \eta_{C}=\frac{T_{2S}-T_{1}}{T_{2}-T_{1}};\;\;\eta_{E}=\frac{T_{4}-T_{5}}{T_{4}-T_{5S}}. \end{array}$$

The following equation is found based on the second law of thermodynamics:

(16) $$\begin{array}{c} T_{1}^{k\;}T_{3}^{1-k\;}T_{4}^{k}=T_{2S\;}T_{5S}^{k}. \end{array}$$

*η*_*C*_ and *η*_*E*_ define the irreversibilities of the adiabatic processes. By using thermodynamic relations between the state points 1–5 and (15)-(16), the following equations are acquired:

(17) $$\begin{array}{c} \left(\frac{T_{2}}{T_{1}}\right)=\frac{\eta_{C}+\left(r^{k-1}-1\right)}{\eta_{C}},\\ \left(\frac{T_{5}}{T_{4}}\right)=\left\{1-\eta_{E}\left[1-{\left(\frac{\rho }{\alpha }\right)}^{k-1}\right]\right\}, \end{array}$$

where the compression ratio (*r*) is given as

(18) $$\begin{array}{c} r=\frac{v_{1}}{v_{2}}. \end{array}$$

Also, the following equation is given based on the second law of thermodynamics [36]:

(19) $$\begin{array}{c} \Delta S_{42}-\Delta S_{51}<0. \end{array}$$

The inequality in (21) could be reordered as follows:

(20) $$\begin{array}{c} I_{\Delta S}\Delta S_{42}-\Delta S_{51}=0\;\text{with}\;\;I_{\Delta S}\geq 1, \end{array}$$

where *I*_Δ*S*_ is internal irreversibility parameter and it is defined as

(21) $$\begin{array}{c} I_{\Delta S}=\frac{\left(S_{5}-S_{1}\right)}{\left(S_{4}-S_{3}\right)+\left(S_{3}-S_{2}\right)}. \end{array}$$

Consequently, the following equation is written as follows:

(22) $$\begin{array}{c} T_{1}^{k\;}T_{3}^{I_{\Delta S}(1-k)\;}T_{4}^{I_{\Delta S}k}=T_{2}^{I_{\Delta S}\;}T_{5}^{k}. \end{array}$$

In the study, dimensionless engine design parameters are the pressure ratio (*β*), cut-off ratio (*ρ*), and source temperature-ratio (*τ*) and they can be given, respectively, as

(23) β=P3P2=T3T2;  ρ=v4v3=T4T3;τ=THTL.

The Atkinson cycle ratio (*r*_*A*_) is derived as Miller cycle ratio [6] and the Atkinson cycle ratio and stroke ratio may be expressed as follows:

(24) $$\begin{array}{c} r_{A}=\frac{v_{5}}{v_{1}}=\frac{T_{5}}{T_{1}};\;\;\alpha =\frac{v_{5}}{v_{2}}=r_{A}r. \end{array}$$

The entropy generation rate of DAC could be written as

(25) $$\begin{array}{c} {\dot{S}}_{g}=\frac{{\dot{Q}}_{LT}}{T_{L}}-\frac{{\dot{Q}}_{HT}}{T_{H}}. \end{array}$$

The ecological objective-function [36] is given as below:

(26) $$\begin{array}{c} \dot{E}=\dot{W}-T_{0}{\dot{S}}_{g}. \end{array}$$

The ECOP criterion is attained as the proportion of the power output to the loss rate of availability as follows [35, 36]:

(27) $$\begin{array}{c} \text{ECOP}=\frac{\dot{W}}{T_{0}{\dot{S}}_{g}}. \end{array}$$

The thermal efficiency may be stated as follows:

(28) $$\begin{array}{c} \eta =\frac{\dot{W}}{{\dot{Q}}_{HT}}. \end{array}$$

The figures are plotted in the next chapter by using the equations given above.

## 3. Results and Discussion

In this section, comprehensive computations are performed evaluating compression ratio (*r*) in order to compare the consequences of DAC depending on different performance parameters such as maximum ECOP, MEF, and MP conditions. The figures in the text are plotted using numerical results. In the calculations, the constants are taken as *k* = 1.4 and *η*_*C*_ = *η*_*E*_ = 0.9 and the total number of heat transfer units is given as below:

(29) $$\begin{array}{c} N_{T}=N_{H1}+N_{H2}+N_{L},\;N_{H1}=N_{H2}. \end{array}$$

**Figure 2:**
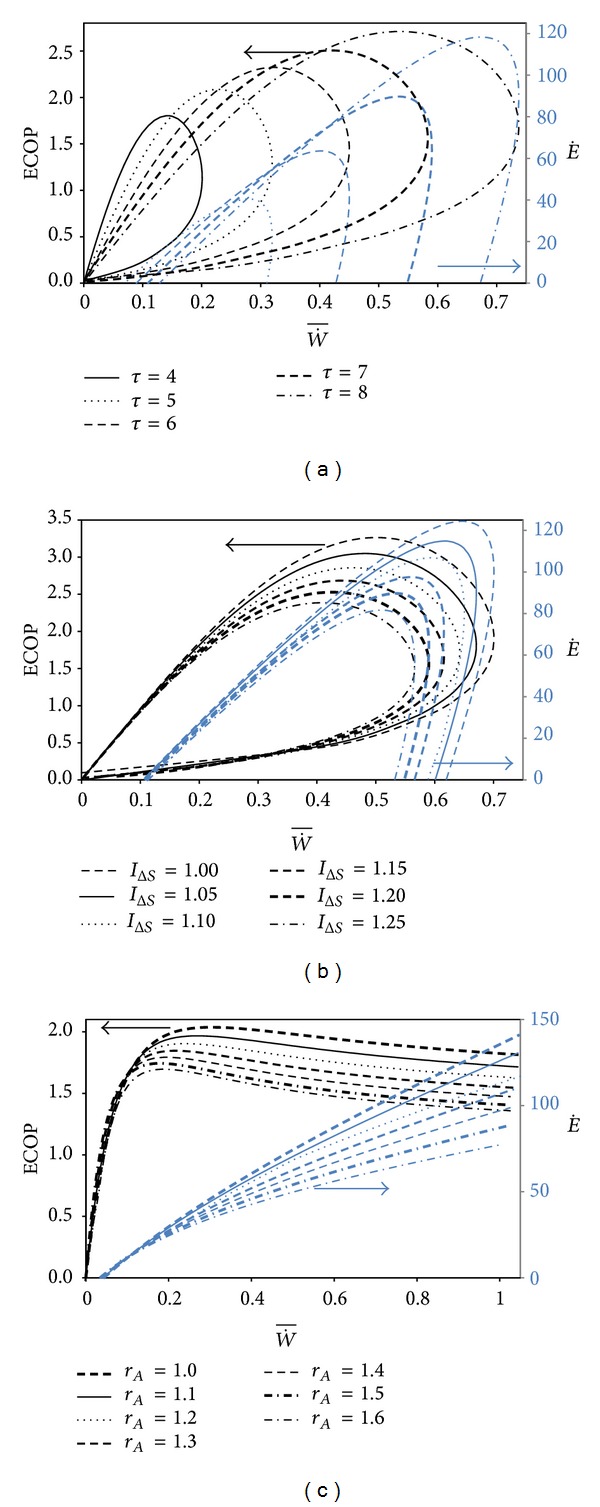
Variations of the ECOP and ecological function with respect to nondimensional power output for (a) various *τ*   (*I*_Δ*S*_ = 1.2); (b) for various *I*_Δ*S*_  (*τ* = 7); (c) for various *r*_*A*_(*I*_Δ*S*_ = 1.2, *α* = 17)  (*T*_0_ = *T*_*L*_ = 300 K, *X* = (*N*_*H*1_ + *N*_*H*2_)/*N*_*T*_ = 0.3, and *ξ* = 0.02).

The variations of the ecological function ($\dot{E}=\dot{W}-T_{0}{\dot{S}}_{g}$) and ECOP with respect to the dimensionless power output ($\bar{\dot{W}}=\dot{W}/{\dot{C}}_{W}T_{L}$) for different *τ*, *I*_Δ*S*_, and *r*_*A*_ are figured in Figures 2(a), 2(b), and 2(c), respectively. Figures 2(a) and 2(b) are plotted with respect to the variation of compression ratio but Figure 2(c) is plotted with respect to variation of *τ*.

It is clearly seen from these figures that the ECOP, *$\dot{E}$*, and $\bar{\dot{W}}$ increase as the source temperature ratio (*τ*) rises and the internal irreversibility parameter (*I*_Δ*S*_) decreases [36]. The Atkinson cycle ratio (*r*_*A*_) increases as the ECOP and *$\dot{E}$* for the same $\bar{\dot{W}}$ values and the maximum $\bar{\dot{W}}$ abates with raising of *r*_*A*_. It is clearly seen from Figure 2(c) that the ECOP increases to a maximum value and then starts to decrease. Nevertheless, *$\dot{E}$* continuously raises with the increase of $\bar{\dot{W}}$. The curves given for the ECOP have more parabolic characteristics although those of *$\dot{E}$* are linear.

It is seen from Figures 2(a) and 2(b) that the maximum ECOP (ECOP_MAX_) is higher than the ECOP at maximum *$\dot{E}$* (ECOP_MEF_) and ECOP at the maximum $\bar{\dot{W}}$ (ECOP_MP_). The order for the ECOP values can be given as ECOP_MP_ < ECOP_MEF_ < ECOP_MAX_. However, the order is ECOP_MP_ = ECOP_MEF_ < ECOP_MAX_ for a specified *r*_*A*_ value.

**Figure 3:**
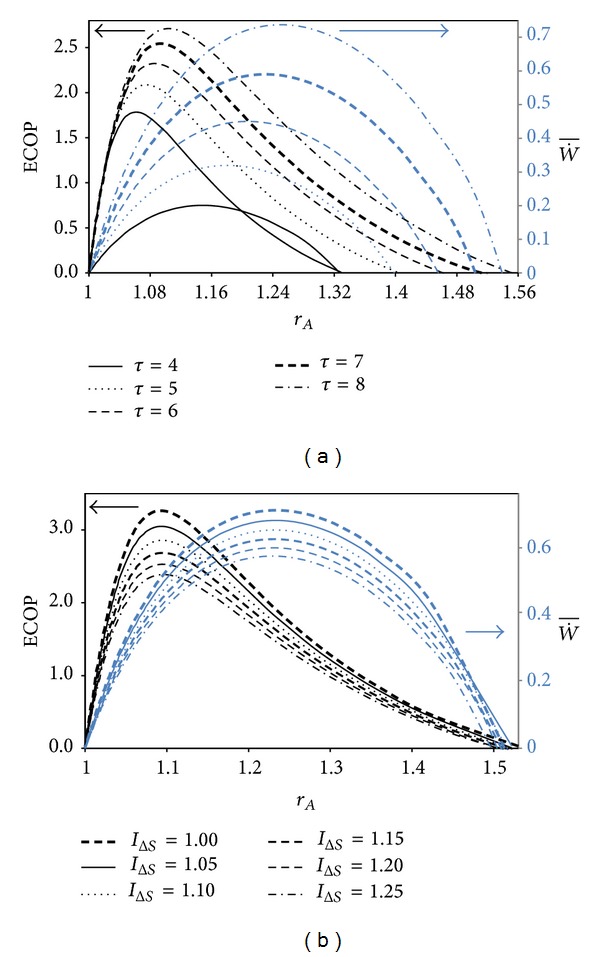
Variations of the ECOP and dimensionless power output with respect to Atkinson cycle ratio for (a) various *τ*, (*I*_Δ*S*_ = 1.2); and (b) for various *I*_Δ*S*_  (*τ* = 7)  (*T*_0_ = *T*_*L*_ = 300 K, *X* = (*N*_*H*1_ + *N*_*H*2_)/*N*_*T*_ = 0.3, and *ξ* = 0.02).

The variations of the ECOP and $\bar{\dot{W}}$ with respect to *r*_*A*_ for different *τ* and *I*_Δ*S*_ are demonstrated in Figures 3(a) and 3(b), respectively. It is observed from the figures that the ECOP and $\bar{\dot{W}}$ raise to a certain value and then begin to abate, while *r*_*A*_ increases. It is obvious that the *r*_*A*_ has optimal values which give the maximum ECOP and $\bar{\dot{W}}$. These optimum values raise while *τ* raises and *I*_Δ*S*_ diminishes. The optimal *r*_*A*_ values for the maximum $\bar{\dot{W}}$ are higher compared to those of the ECOP in all conditions.

**Figure 4:**
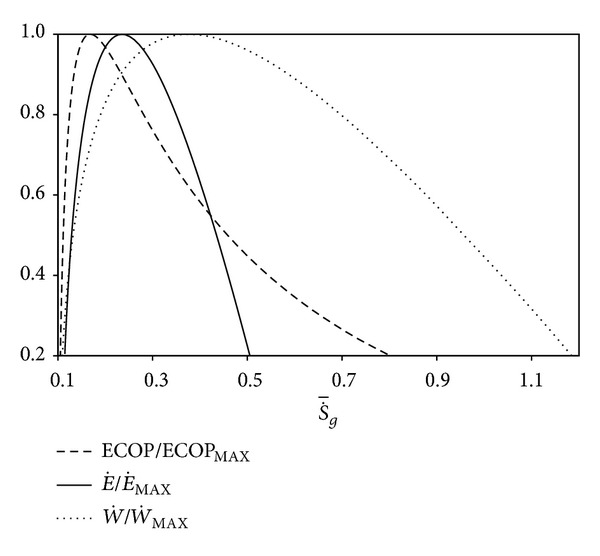
Variations of the normalized ECOP, $\bar{\dot{W}}$ and *$\dot{E}$* with respect to dimensionless entropy generation rate (*T*_0_ = *T*_*L*_ = 300 K, *I*_Δ*S*_ = 1.2, *τ* = 7, *X* = 0.3, and *ξ* = 0.02).

Normalized forms of the ECOP, dimensionless power output, and ecological function with respect to the dimensionless entropy generation rate (${\overset{-}{\dot{S}}}_{g}={\dot{S}}_{g}/{\dot{C}}_{W}$) are depicted in Figure 4. ${\overset{-}{\dot{S}}}_{g_{\text{MP}}}$, ${\overset{-}{\dot{S}}}_{g_{\text{MEF}}}$, and $\overset{-}{{\dot{S}}_{g}^{*}}$ stand for the dimensionless entropy generation rate at the maximum dimensionless power output (${\bar{\dot{W}}}_{\text{MAX}}$), at the maximum ecological function (${\dot{E}}_{\text{MAX}}$), and at the maximum ECOP (ECOP_MAX_), respectively. The order may be expressed as $\overset{-}{{\dot{S}}_{g}^{*}}<{\overset{-}{\dot{S}}}_{g_{\text{MEF}}}<{\overset{-}{\dot{S}}}_{g_{\text{MP}}}$. This result is similar to that of [36].

**Figure 5:**
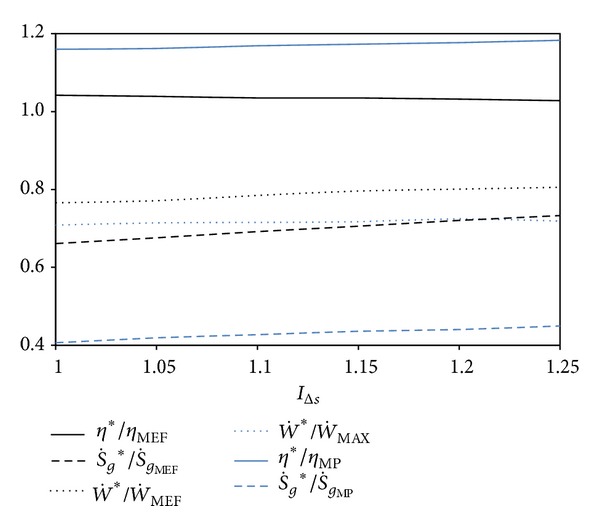
Comparisons of the optimum performances at ECOP_MAX_, ${\dot{E}}_{\text{MAX}}$, and ${\dot{W}}_{\text{MAX}}$ conditions based on *I*_Δ*S*_ for *τ* = 7  (*T*_0_ = *T*_*L*_ = 300 K, *X* = 0.3, and *ξ* = 0.02).

Figure 5 shows the comparison of the performance parameters of *η*, $\dot{W}$, and ${\dot{S}}_{g}$ for ECOP_MAX_, ${\dot{E}}_{\text{MAX}}$, and ${\dot{W}}_{\text{MAX}}$ conditions. It is understood from the figures that the ECOP_MAX_ circumstances are more advantageous compared to ${\dot{E}}_{\text{MAX}}$ circumstances in point of ${\dot{S}}_{g}$ and thermal efficiency (*η*), however, contrarily, disadvantageous compared to that with regard to $\dot{W}$circumstances.

We see from the figures that the ECOP_MAX_ conditions for the entropy generation rate and thermal efficiency and the disadvantage for the dimensionless power output raise while *I*_Δ*S*_ reduces. The results show that ECOP_MAX_ circumstances are more advantageous over ${\dot{E}}_{\text{MAX}}$ circumstances up to 35%, 4% and disadvantageous up to 33% in points of ${\dot{S}}_{g}$, *η*, and $\dot{W}$, respectively; ECOP_MAX_ circumstances are more advantageous over ${\dot{W}}_{\text{MAX}}$ circumstances up to 58%, 22% and disadvantageous up to 29% in points of ${\dot{S}}_{g}$, *η* and $\dot{W}$, respectively. It should be noted that the variation of *I*_Δ*S*_ remarkably affects ${\dot{E}}_{\text{MAX}}$ circumstances, even though it has no notable influence on ${\dot{W}}_{\text{MAX}}$ circumstances.

**Figure 6:**
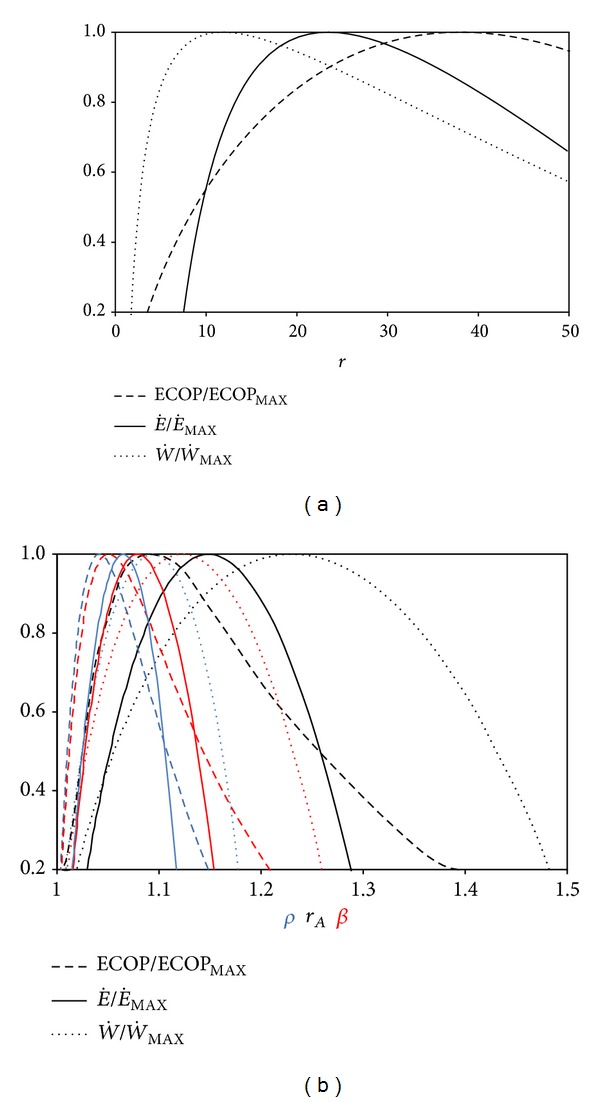
Normalized ECOP*$\dot{E}$* and $\dot{W}$ versus (a) *r* and (b) *ρ*, *r*_*A*_, *β*  (*T*_0_ = *T*_*L*_ = 300 K, *τ* = 7, *I*_Δ*S*_ = 1.2, *X* = 0.3, and *ξ* = 0.02).

The substantial nondimensional engine design parameters are *r*, *r*_*A*_, *β*, and *ρ*. The results of the analysis based on these parameters are illustrated in Figures 6(a) and 6(b). It is clear that *r* at ${\dot{W}}_{\text{MAX}}$ circumstances (*r*_MP_) is smallest compared to those at ECOP_MAX_ (r*) and ${\dot{E}}_{\text{MAX}}$ (*r*_MEF_) circumstances. Hence, the relation between the optimal compression ratios for different conditions may be expressed as r* > *r*_MEF_ > *r*_MP_. On the other hand, the relation between Atkinson cycle ratios (*r*_*A*_), pressure ratios (*β*), and cut-off ratios (*ρ*) is just the opposite as may be seen from Figure 6(b). The order between these parameters could be stated as *r*_A_* < *r*_*A*,MEF_ < *r*_*A*,MP_, β* < *β*_MEF_ < *β*_MP_, and ρ* < *ρ*_MEF_ < *ρ*_MP_. Also, it may be observed that the order is *ρ* < *r*_*M*_ < *β* at same maximum conditions.

**Figure 7:**
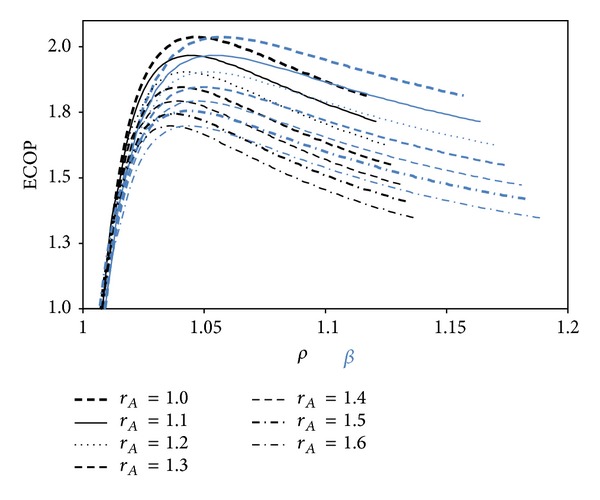
ECOP versus *ρ*, *β* for different Atkinson cycle ratios (*I*_Δ*S*_ = 1.2, *α* = 17)  (*T*_0_ = *T*_*L*_ = 300 K, *X* = 0.3, and *ξ* = 0.02).

Figure 7 demonstrates the variation of the ECOP with respect to the *ρ* and *β* for different Atkinson cycle ratios. The ECOP abates, while *r*_*A*_ raises for the same *ρ* and *β* values. It may be also seen from the figure that the maximum *ρ* is smaller than *β* at the same *r*_*A*_ values.

**Figure 8:**
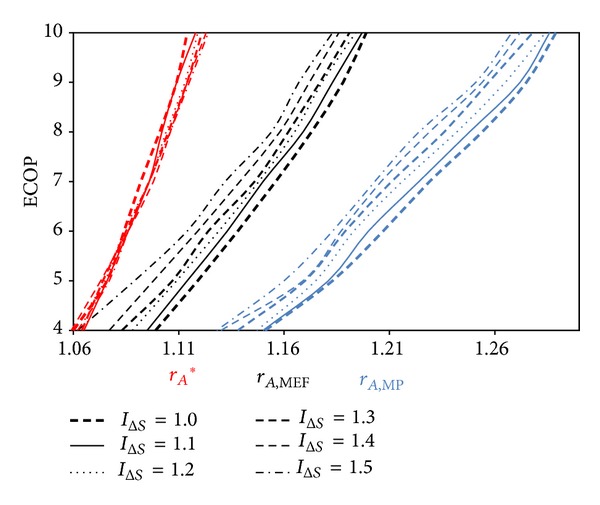
*τ* versus *r*_*A*_ at ECOP_MAX_, ${\dot{E}}_{\text{MAX}}$, and ${\dot{W}}_{\text{MAX}}$ circumstances for different *I*_Δ*S*_ values  (*T*_0_ = *T*_*L*_ = 300 K, *α* = 17*X* = 0.3, and *ξ* = 0.02).

Figure 8 illustrates the variation of *r*_*A*_ values with respect to *τ* for different *I*_Δ*S*_ values. *r*_*A*_ values at ECOP_MAX_ (*r*_A_*) are smaller than those at ${\dot{E}}_{\text{MAX}}$ (*r*_*A*,MEF_) and ${\dot{W}}_{\text{MAX}}$ (*r*_*A*,MP_); this relationship is similar to those of *ρ* and *β*. The relation can be written as *r*_A_* < *r*_*A*,MEF_ < *r*_*A*,MP_. As may be observed from the figure, *r*_*A*_ values at the maximum conditions raise with increasing of *τ*. For specified *τ*, *r*_*M*,MEF_ reduces steadily, whilst *I*_Δ*S*_ raises. Nevertheless, the variation of *r*_M_* and *r*_*M*,MP_ is unstable with the variation of *I*_Δ*S*_.

**Figure 9:**
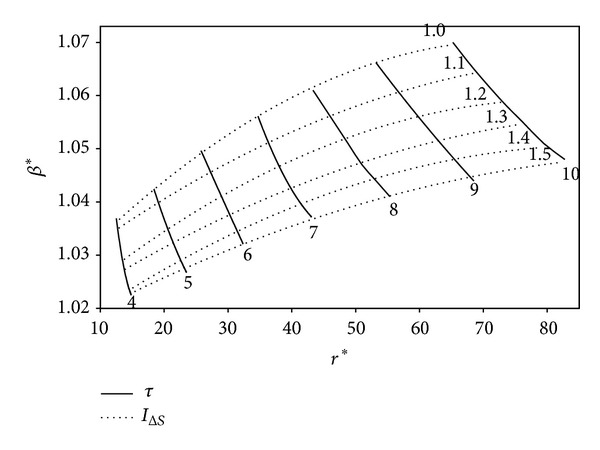
Variation of the optimum *β* at ECOP_MAX_ circumstances based on the optimum *r*, for various *τ* and *I*_Δ*S*_ values (*T*_0_ = *T*_*L*_ = 300 K, *X* = 0.3, and *ξ* = 0.02).

**Figure 10:**
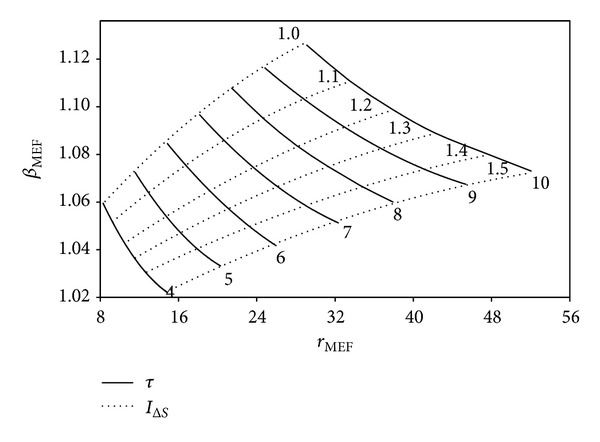
Variation of the optimum *β* at ${\dot{E}}_{\text{MAX}}$ circumstances based on the optimum *r*, for various *τ* and *I*_Δ*S*_ values (*T*_0_ = *T*_*L*_ = 300 K, *X* = 0.3, and *ξ* = 0.02).

**Figure 11:**
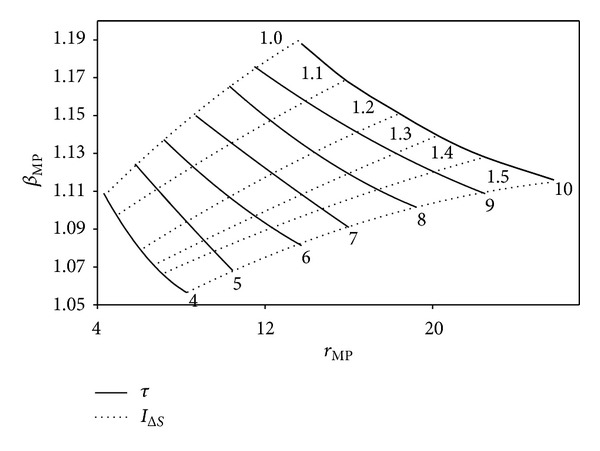
Variation of the optimum *β* at ${\dot{W}}_{\text{MAX}}$ circumstances based on the optimum *r*, for various *τ* and *I*_Δ*S*_ values (*T*_0_ = *T*_*L*_ = 300 K, *X* = 0.3, and *ξ* = 0.02).

The impacts of *I*_Δ*S*_ and *τ* on the variation of the optimum *r* and *β* in terms of ECOP_MAX_, ${\dot{E}}_{\text{MAX}}$, and ${\dot{W}}_{\text{MAX}}$ are illustrated in Figures 9, 10, and 11, respectively. It is seen from the figures that the optimum values of the engine design parameters increase as *τ* raises for a determined *I*_Δ*S*_ value at the ECOP_MAX_, ${\dot{E}}_{\text{MAX}}$, and ${\dot{W}}_{\text{MAX}}$ conditions. One can see that *β* reduces and *r* raises with the increase of *I*_Δ*S*_ for a specified *τ* value. However, increase rate of *r*_*A*,MEF_ and *r*_*A*,MP_ is higher compared to that of *r*_M_*. Therefore, the optimum values of the engine design parameters change with respect to the determined objective-function, for a specified *τ*. The maximum and minimum values of β*, *β*_MEF_, and *β*_MP_ are 1.08; 1.13; 1.19 and 1.026; 1.03; 1.054, respectively. The maximum and minimum values of r*, *r*_MEF_, and *r*_MP_ are 83; 52; 26 and 12; 8; 4, respectively. It is clear that there is reverse relation for the optimum *r* values; the orders may be given as r* > *r*_MEF_ > *r*_MP_, β* < *β*_MEF_ < *β*_MP_.

## 4. Conclusion

A thermoecological performance analysis has been conducted so as to define the optimum engine operation and design parameters for the air-standard irreversible DAC cycle having a finite-rate of heat transfer, heat leakage and internal irreversibilities based on the ecological function, the maximum power output, and the ECOP criteria.

In this standpoint, the optimum pressure ratio (*β*), cut-off ratio (*ρ*), compression ratio (*r*), and the Atkinson cycle ratio (*r*_*A*_) that maximize the ecological coefficient of performance (ECOP_MAX_), ecological function (${\dot{E}}_{\text{MAX}}$), and power output (${\dot{W}}_{\text{MAX}}$) have been examined. And also a comparative study based on maximum values of these criteria has been realized for an irreversible Atkinson cycled engine model; the relations between the power output and ECOP and the Atkinson cycle ratio and ECOP for different *τ* and *I*_Δ*S*_ values have been derived.

The ECOP, *$\dot{E}$*, and $\bar{\dot{W}}$ versus *ρ* and *β* figures for the DAC have been illustrated for *τ* and *I*_Δ*S*_ values. It is noted that ECOP_MAX_ circumstances have substantial vantage over the ${\dot{E}}_{\text{MAX}}$ circumstances in point of entropy formation rate and little vantage in point of thermal efficiency: on the other hand, a slight disadvantage is observed in point of power output.

Comparisons at the maximum power output conditions (${\overset{-}{\dot{W}}}_{\text{MAX}}$) show that ECOP_MAX_ circumstances have significant vantages in terms of ecological viewpoints with little loss of power output. The optimum *r*_*A*_, *r*, *β*, and *ρ* values at ECOP_MAX_, ${\dot{E}}_{\text{MAX}}$, and ${\bar{\dot{W}}}_{\text{MAX}}$ conditions with respect to the variation of *τ* and *I*_Δ*S*_ have been depicted so as to provide good guidelines for the description of the optimal design and operating conditions of real Atkinson cycle diesel engines.

## Nomenclature

*A*: Heat transfer area (m^2^)
*C*_*P*_: Specific heat at constant pressure (kW/kg · K)
${\dot{C}}_{W}$: $\dot{m}C_{V}\;\;\left(\text{kW}/\text{K}\right)$
DAC: Dual-Atkinson cycle
*$\dot{E}$*: Ecological performance function
ECOP: Ecological coefficient of performance
*I*_Δ*S*_: Internal irreversibility parameter
*k*: Isentropic exponent
$\dot{m}$: Mass flow rate (kg/s)
*N*_*T*_: Total number of heat transfer units
*P*: Pressure (kPa)
$\dot{Q}$: Rate of heat transfer (kW)
*r*: Compression ratio *r* = *v*_1_/*v*_2_
*r*_*A*_: Atkinson cycle ratio, *r*_*A*_ = *v*_6_/*v*_1_
*S*: Entropy (kJ/K)
*T*: Temperature (K)
*U*: Overall heat transfer coefficient (kW/m^2^K)
*V*: Volume (m^3^)
$\dot{W}$: Power output (kW).

### Greek Letters

*α*: Stroke ratio, *α* = *v*_6_/*v*_2_
*β*: Pressure ratio, *β* = *T*_3_/*T*_2_
*ε*: Heat-exchanger effectiveness
*χ*: Allocation ratio (*N*_*H*1_ + *N*_*H*__2_)/*N*_*T*_
*η*: Thermal efficiency
*η*_*C*_: Isentropic efficiency of compression
*η*_*E*_: Isentropic efficiency of expansion
*ρ*: Cut-off ratio, *ρ* = *T*_4_/*T*_3_
*τ*: Source temperature ratio *τ* = *T*_*H*_/*T*_*L*_.

### Subscripts

*g*: Generation
*H*: High-temperature heat-source
*L*: Low-temperature heat-source
MAX: Maximum
MEF: At the maximum ecological objective-function condition
MP: At maximum power output condition
0: Environment condition.

### Superscripts

*: At the maximum ECOP condition
—: Dimensionless.
